# Level and timing of physical activity during normal daily life in depressed and non-depressed individuals

**DOI:** 10.1038/s41398-020-00952-w

**Published:** 2020-07-30

**Authors:** Olga Minaeva, Sanne H. Booij, Femke Lamers, Niki Antypa, Robert A. Schoevers, Marieke Wichers, Harriëtte Riese

**Affiliations:** 1 Interdisciplinary Center for Psychopathology and Emotion Regulation, Department of Psychiatry, University Medical Center Groningen, University of Groningen, Groningen, The Netherlands; 2 Department of Developmental Psychology, Faculty of Behavioural and Social Sciences, University of Groningen, Groningen, The Netherlands; 3 Center for Integrative Psychiatry, Lentis, Groningen, The Netherlands; 4 Department of Psychiatry, Amsterdam Public Health Research Institute, Amsterdam UMC, Vrije Universiteit, Amsterdam, The Netherlands; 5 Department of Clinical Psychology, Institute of Psychology, Leiden University, Leiden, The Netherlands

**Keywords:** Depression, Human behaviour

## Abstract

Engaging in physical activity is known to reduce depressive symptoms. However, little is known which behavioral factors are relevant, and how patterns of activity change during depressive episodes. We expected that compared to controls, in depressed individuals the level of activity would be lower, the amplitude of 24-h-actigraphy profiles more dampened and daytime activities would start later. We used 14-day continuous-actigraphy data from participants in the Netherlands Study of Depression and Anxiety (NESDA) who participated in an ambulatory assessment study. Participants with a depression diagnosis in the past 6 months (*n* = 58) or its subsample with acute depression (DSM diagnosis in the past 1 month, *n* = 43) were compared to controls without diagnoses (*n* = 63). Depression was diagnosed with a diagnostic interview. Actigraphy-derived variables were activity mean levels (MESOR), the difference between peak and mean level (amplitude) and the timing of the activity peak (acrophase), which were estimated with cosinor analysis. Compared to the control group, both depression groups (total: *B* = −0.003, *p* = 0.033; acute: *B* = −0.004, *p* = 0.005) had lower levels of physical activity. Amplitude was also dampened, but in the acute depression group only (total: *B* = −0.002, *p* = 0.065; acute: *B* = −0.003, *p* = 0.011). Similarly, the timing of activity was marginally significant towards a later timing of activity in the acute, but not total depression group (total: *B* = 0.206, *p* = 0.398; acute: *B* = 0.405, *p* = 0.084). In conclusion, our findings may be relevant for understanding how different aspects of activity (level and timing) contribute to depression. Further prospective research is needed to disentangle the direction of the association between depression and daily rest-activity rhythms.

## Introduction

Large epidemiological studies indicate that physical activity (PA) is negatively associated with the prevalence of depressive disorders and severity of depressive symptoms^1–3^, and that exercise can improve depressive symptoms in nonclinical^4,5^ and clinical populations^6,7^. Besides effects of altered levels of PA on depression, changes in diurnal rhythm activity patterns, which include chronobiological factors, may be involved. Unraveling the different aspects of PA that tap into depression may be helpful for further understanding of the pathogenesis of depression and for future practical implementation. Therefore, we will examine these two possibilities using unique data from 2-week continuous actigraphy in both depressed and healthy individuals.

Prior studies examined the association between PA levels and depressive symptoms and disorders. The effect is mostly explained as that more PA generates positive emotions^8^, or has a favorable, neurotrophic impact on the brain^9^. In line with this, lower levels of activity were found to predispose to affective depressive symptoms, such as depressed mood and anhedonia, which are the core symptoms of depressive disorders^10^. Also, healthy individuals with low self-reported PA were at risk for developing depression, while higher PA levels were protective against developing depression among older adults^11^ and a general population^12^. Both studies using self-report measures of the amount of PA, as well as studies with objective actigraphy measures, confirmed the negative association between activity and depression^13–18^.

While prior actigraphy studies provided valuable insights, they have several limitations. These studies were conducted in small samples (40–54 participants)^13,15,16^, mostly used relatively short assessment periods (i.e. between 12 h and 7 days)^13,15,17^, and examined specific subgroups of patients^14,17^, with the notable exception of the study of Difrancesco et al.^18^. The latter study conducted 14 days of actigraphy measurements in a large sample of participants (*n* = 359). However, more than 7 days of measurement are needed to obtain reliable estimates of PA for most variables of interest^19^. A recent study reported a reduced overall level of PA among individuals with either anxiety or depression episode in the last 6 months compared to remitted and healthy controls^18^. A substantive point in how this current study is different from previous ones, however, is the investigation of the timing of activity together with the level of activity.

Physical activity may be associated with depression due to changes in diurnal activity rhythm. The circadian rhythm, which regulates various physical, mental, and behavioral changes during a 24-h cycle^20^, can be advanced or delayed in depressed individuals with regards to the timing of activity^21^ or dampened^13^. This may cause shifting in the preferred timing of PA in depressed individuals^22,23^, possibly expressed as sleep problems, changed sleep duration^24^ or less exposure to beneficial daylight^25^. Therefore, the timing of PA may be an important factor in the association between PA and depression. Further support for this assumption comes from studies on chronotype that report the association between evening type and more depressive symptoms^26–29^ or depression^30–32^.

Among actigraphy studies on the timing of daily PA, one showed that the later timing of PA is associated with increased risk of developing depressive symptoms among elderly men^14^. Two other studies, in elderly women^33^ and in a small group of outpatients^13^, did not find a significant association with the timing of PA, but only a tendency towards a later timing among depressed individuals. Although studies mentioned above have provided insight in the timing of activity, their findings are inconclusive and mostly focused on specific age and population groups^14,33^.

Currently, it is unclear whether associations between PA and depression originate only from a lower level of PA, or whether this association additionally arises from shifted timing of PA due to a change in diurnal rhythm. The latter was even less studied using actigraphy measurements in depressed populations. As it is important to know what behavioral factors are at play in order to optimize targets for intervention, we will study both the level and timing of activity. The current study also complements previous studies by simultaneously using objective measures of both the level and timing of PA, measured by continuous actigraphy over 2 weeks. We use partly the same data as Difrancesco et al.^18^, but focus specifically on individuals with depressive disorders, excluding individuals with pure anxiety disorders. Additionally, we will focus on individuals with an acute diagnosis (within the past month), on top of individuals with diagnosis of depression in the past 6 months. Studying a more homogeneous (i.e. acutely), depressed group allows us to examine acute symptoms and not merely residual symptoms during recovery from latter depressive episodes.

We also use a fine-grained methodology, which allows us to look at diurnal activity patterns with a longer assessment period than most previous studies and in both depressed and healthy individuals. In the current study, we hypothesized (1) that depressed individuals have lower levels of PA than controls, (2) depressed individuals have dampened diurnal rhythms compared to healthy controls, and (3) become active later during the day due to a shift in their circadian phase.

## Materials and methods

### Participants

We used data from the Netherlands Study of Depression and Anxiety (NESDA, www.nesda.nl). Details about NESDA were given extensively before^34^. In short, NESDA is an ongoing multisite naturalistic longitudinal cohort study among 2981 adults (18–65 years) at baseline, including individuals with depressive disorders and/or anxiety disorders, as well as healthy controls, which were recruited from community, primary care, and specialized mental health-care settings. Patients with a psychotic disorder, bipolar disorder, obsessive–compulsive disorder, or substance use disorder, and persons not fluent in Dutch were excluded from the baseline assessment. The NESDA study was approved by the VUmc ethical committee (reference number 2003/183) and all respondents gave informed consent.

In the current study, we used a subsample (*n* = 121) from wave 6 (9-year follow-up), which contained a 14-day period of ecological momentary assessments. Details of the actigraphy measurements are given elsewhere^18^. Of the 384 initially included participants, 14 had no available actigraphy data for several reasons, such as technical failure, 10 individuals had less than 1 weekday or weekend-day data available. For this study, we examined the effect of a depressive episode; therefore, participants with remitted depressive disorder (*n* = 152), with anxiety disorder only (*n* = 67) and the siblings of NESDA participants (*n* = 20) were excluded. As comorbidity of anxiety and depressive disorders is common^35^, this was not an exclusion criterion. Actigraphy data from individuals with an episode of depressive disorder (major depressive disorder and/or dysthymia) in the past 6 months (called “total depression group”, *n* = 58) and from individuals with no lifetime depressive and anxiety disorder (controls, *n* = 63) were used. Additionally, we created a subgroup of the total depression group consisting of people with a depressive episode in the past month (called “acute depression group”, *n* = 43).

### Actigraphy

Participants completed 14 consecutive days of all-day actigraphy activity monitoring. They wore a GENEActiv actigraphy device on a nondominant wrist. Participants were instructed to wear the watch day and night and only taking it off when going to the sauna or when playing a contact sport in which wearing a wristband is unsafe. The device is a triaxial wrist accelerometer recording data in SI units represented as acceleration in three axes (*x*, *y*, and *z*). GENEActiv validity studies have demonstrated strong correlations for criterion validity (Pearson *r* = 0.79–0.98)^36^ and a good ability to determine sedentary behavior in adults (18–55 years) (Pearson *r* = 0.81)^37^. To pre-process the raw data, an open-source R package, GGIR (version 1.5-12)^38^ was used. At the first stage of pre-processing, we verified sensor calibration error using local gravity as a reference, checked for abnormally high values, non-wear periods, and then extracted objective PA measures. During raw data cleaning, missing data (e.g. suspected of monitor non-wear) or invalid data were imputed by the averages at similar time points on different days of the week, as GGIR does by default. In this way, within-day variability of the data should not be affected, unlike the situation when the data are imputed with the average of close by time points of the same day. Only respondents with valid actigraphy data for at least 16 h per day, and at least 10 days were included in the analysis (*n* = 121). To estimate PA, we calculated a metric ENMO (Euclidean Norm Minus One) using the formula $\sqrt {x^2 + y^2 + z^2} - 1g$ with any negative values rounded up to zero and by reducing such measure over 1-min epochs^39^. We adapted and executed the R-script for the current study.

### Predictor and outcome measures

The main predictor in the study was clinical group status (depression diagnosis versus healthy control status). Diagnoses were acquired with the Composite International Diagnostic Interview (CIDI, version 2.1) based on the DSM-IV^40^. The CIDI has high interrater reliability, high test–retest reliability, and high validity for depressive disorders^40^. Participants were divided into the following groups based on the results of a CIDI assessment: (1) a group with no lifetime history of psychiatric disorders (healthy controls) (*n* = 63), and (2) a group with a depressive disorder (either with or without anxiety comorbidity in the past 6 months, *n* = 58). In a next step, to assess whether there was an acute state effect of depression on PA, we selected a subgroup of the 6-month depressed group with a depressive disorder present in the past 1 month (termed “acute”, *n* = 43) in the analysis to compare to the control group.

The main outcome variables were three actigraphy-derived PA variables: mean levels of activity (MESOR, an acronym for Medline Estimated Statistic of Rhythm), height of peak with respect to the mean level (amplitude), and the timing of the activity peak (acrophase). MESOR, as a measure of the mean level of the curve between the highest and the lowest point, represents the average activity level of the day. Amplitude, as a measure of the difference between activity during the day and the night, indicates the robustness of the diurnal rhythm. Acrophase, as a measure of the time of the day when the activity peak occurred, indicates timing preferences of an individual during the day. MESOR and amplitude were measures for the level of PA, and acrophase—a measure for the timing of PA. To calculate these variables, circadian rhythms were estimated by fitting individual 1-min epoch ENMO data to a cosine curve of a 24-h activity rhythm, which was obtained by the cosinor method^41,42^ using the following equation: *y*(*t*) *=M+A*cos(2*πt/τ+ϕ*) *+e*(*t*), where *y* is the selected variable, *t* is the elapsed time, *M* is MESOR, a rhythm-adjusted mean, *A* is the amplitude of circadian rhythm, *τ* is the period of the circadian rhythm (the 24-h period), *ϕ* is the acrophase, and *e*(*t*) is the error term.

Cosinor analysis was performed in R statistical software version 1.1.383^43^. For this purpose, we created an R script, which obtains daily values (i.e. 14 values per person) of the cosinor parameters MESOR, amplitude, and acrophase, and automates this process across persons and days and exporting the values to a new data set. The script is given in Supplementary Materials 1.

### Chronotype

Chronotype was assessed with the Munich Chronotype Questionnaire (MCTQ)^44^. The MCTQ is not a scale; therefore, its reliability cannot be assessed. The MCTQ highly correlates with the Morningness−Eveningness Questionnaire (MEQ) (*r* = –0.73)^45^, which in turn, consistently report high levels of reliability (>0.80)^46^. Chronotype was defined as the midpoint in time between falling asleep and waking up on free days corrected for “oversleep” due to the sleep debt that individuals accumulate over the workweek (MSFsc).

### Potential confounding variables

Demographics (gender, age, marital status), socioeconomic status (education, employment)^12^, smoking status, BMI^47^, current medication use (e.g. benzodiazepine, antidepressants)^13^, and depression severity were included in the analysis as additional covariates. Depression severity was assessed with the Inventory of Depressive Symptomatology (IDS)^48^. Internal consistency of the IDS-SR is high, and the Cronbach α ranged from 0.67 to 0.94^48,49^. Current medication use was based on drug container inspection and medications were coded according to the World Health Organization Anatomical Therapeutic Chemical (ATC) classification and considered present if participants reported currently using psychopharmacological medication.

### Statistical analysis

Baseline characteristics were compared pairwise between the control group and the total depression (diagnosis in the past 6 months) or acute depression (diagnosis in the past 1 month) group, respectively. The independent *t* test was used for continuous variables (age, BMI, education, IDS scores) and Chi-squared test was used for categorical variables (gender, marital status, employment, smoking status, IDS severity groups, and psychopharmaca use).

As the data are hierarchically structured, we used multilevel linear modeling to assess whether the MESOR, amplitude, and acrophase of the PA rhythm differed between individuals with and without the diagnosis of depression. A two-level data structure was used where days of actigraphy were defined as the first-level unit and individuals as the second-level unit. The first set of analyses was performed using the 6-month cut-off for an episode of depressive disorder (total depression group). Two models were run for each outcome variable. The first model assessed the association between group (non-depressed = 0, depressed = 1) and PA levels and timing (Model 1). Next, this association was tested while including all covariates (Model 2). Only for acrophase, a third model (Model 3) was tested as an additional check whether chronotype (also an indicator of the phase of the circadian rhythm) explains (part of) the association between acrophase and group status. A significant association would support the hypothesis that a shift in acrophase indeed reflects a shift in the circadian rhythm.

For the second set of analyses, all models for three outcome variables (MESOR, amplitude, and acrophase) were repeated in the subsample of acutely depressed (1-month cut-off, *n* = 43) versus controls.

Besides the fixed effects (group status and the covariates), a random intercept and time trend (day number) were included if they improved the model fit. Model fit was assessed using the Akaike Information Criterion (AIC) (lower is better). For all models, a random intercept, but not a slope for day number, improved the model fit. The best fitting covariance structure for the random effects was the variance components (VC) structure. In addition, to correct for potential autocorrelation of the residuals, which is often present in repeated assessments data, we examined several possible covariance structures for the residuals. The best structure was an autoregressive heterogeneous (AR(1)H). Multilevel analyses were conducted using SPSS Statistics version 25. Significance levels were set at *p* < 0.05.

The residuals of the final models were not normally distributed. To overcome this issue, we repeated all analyses with a bootstrap approach, as bootstrapping is robust against violations of normality^50^. Due to model complexity, we needed to slightly adapt the model; instead of specifying an AR(1)H covariance structure for the residuals, we included a lag(1) outcome variable as a fixed and random predictor. This way, potential autocorrelation was still addressed. Even though the bootstrap analysis gave similar results, and in three cases borderline significant findings even became significant, we decided to keep a more conservative approach to prevent overstating the results. Therefore, we reported the results of the original models only. However, the syntax and the results of the bootstrap analyses are given in the Supplementary Materials 2–4 (Tables S1 and S2).

## Results

### Sample description

Demographic and clinical characteristics of the participants are given in Table 1. Participants (*n* = 121) have a mean age of 52.13 (SD = 11.3; range 28−72) years, and 63.6% were women. No significant differences in age, gender, marital status, BMI, and chronotype were found between the total depression group and controls. Depressed individuals were more often unemployed and smokers, had lower education, higher mean depressive symptom scores, and used more medication compared to the control group. The comparisons of the acute depression (*n* = 43) group to the control group gave similar results. Mean values for circadian rest-activity characteristics (MESOR, amplitude, and acrophase) across groups are given in Table 2. As expected, effect sizes are somewhat larger in the 1-month depression group than in the 6-month depression group regarding all three outcome variables.

**Table 1:** Demographic and clinical characteristics of depressed patients and controls (*n* = 121).

	Total depression group (6 months)	Acute depression group (1 month)^a^	Control group	*p* value	
	(*n* = 58)	(*n* = 43)	(*n* = 63)	6m/control^b^	1m/control^c^
Demographics					
Age, years: mean (SD)^d^	52.34 (10.59)	52.14 (9.57)	51.94 (12.05)	0.844	0.927
Gender, female: *n* (%)^e^	36 (62.10)	29 (67.40)	41 (65.10)	0.850	0.837
Education, years: mean (SD)^d^	12.17 (3.16)	11.81 (3.16)	13.94 (2.82)	**0.002**	**<0.001**
Marital status, married: *n* (%)^e^	28 (48.30)	22 (51.20)	36 (57.10)	0.194	0.396
Employment, yes: *n* (%)^e^	22 (38.00)	15 (34.90)	48 (76.20)	**<0.001**	**<0.001**
Lifestyle					
Smoking status, yes: *n* (%)^e^	20 (34.50)	17 (39.50)	7 (11.10)	**0.020**	**0.007**
Body mass index (BMI): mean (SD)^d^	27.36 (4.92)	27.48 (5.07)	26.06 (5.57)	0.178	0.181
Psychiatric disorders					
Only depressive disorders: *n* (%)	7 (12.10)	1 (2.30)	0(0)	—	—
Depressive and anxiety disorders: *n* (%)	51 (87.90)	42 (97.70)	0(0)	—	—
Psychological scales					
IDS, raw score: mean (SD)^d,f^	30.34 (13.10)	34.53 (11.73)	5.02 (3.00)	**<0.001**	**<0.001**
IDS, severity: *n* (%)^e^					
None	8 (13.80)	3 (7.00)	63 (100)	**<0.001**	**<0.001**
Mild	11 (19.00)	4 (9.30)	—		
Moderate	25 (43.10)	22 (51.20)	—		
Severe	9 (15.50)	9 (20.90)	—		
Very severe	5 (8.60)	5 (11.60)	—		
Chronotype					
MSFsc, h: mean (SD)^d,g^	3.63 (1.70)	3.60 (1.71)	3.61 (1.35)	0.956	0.945
Current medication use					
Psychopharmaca users: *n* (%)^e^	30 (51.70)	23 (53.50)	3 (4.80)	**<0.001**	**<0.001**
Antidepressants: *n* (%)	27 (46.60)	20 (86.90)	0 (0)		
Benzodiazepines: *n* (%)	7 (12.10)	6 (26.10)	1 (1.60)		
Antipsychotics: *n* (%)	4 (6.90)	4 (17.40)	0 (0)		
Lithium: *n* (%)	2 (3.40)	1 (4.30)	0 (0)		
Other types^h^: *n* (%)	0 (0)	0 (0)	2 (3.20)		

*IDS* Inventory of Depressive Symptomatology, *6m* 6-month depression group, *1m* 1-month depression group, *MSFsc* mid-sleep time on free days corrected for sleep debt on workdays.

^a^The subgroup of the total depression group.

^b^*p* values for the comparison between the total depression group (6 months) and control group.

^c^*p* values for the comparison between the acute depression group (1 month) and control group.

^d^Independent *t* test.

^e^Chi-squared test (two-sided).

^f^Depression Severity: 0–13—None, 14–25—Mild, 26–38—Moderate, 39–48—Severe, 49–84—Very severe.

^g^Chronotype differences were not significant and therefore interpreted as negligible across the groups.

^h^Anticonvulsants and smoking cessation medication.

*P*-values at or below 0.05 are marked in bold.

**Table 2:** Mean values for MESOR, acrophase and amplitude on a person level across groups (*n* = 121).

Characteristic	Total depression group	Acute depression group^a^	Control group	Cohen’s *d*	
				1/0^b^	2/0^b^
MESOR, mean (SD)	0.024 (0.007)	0.023 (0.007)	0.027 (0.006)	0.500	0.657
Amplitude, mean (SD)	0.020 (0.007)	0.019 (0.006)	0.022 (0.006)	0.357	0.503
Acrophase, mean (SD)	14.757 (1.122)	14.860 (1.180)	14.604 (0.918)	0.150	0.244

^a^The subgroup of the total depression group.

^b^1/0—total depression group compared to controls; 2/0—acute depression group compared to controls.

### Actigraphy differences between depressed participants (6-month cut-off) and controls

**Fig. 1:**
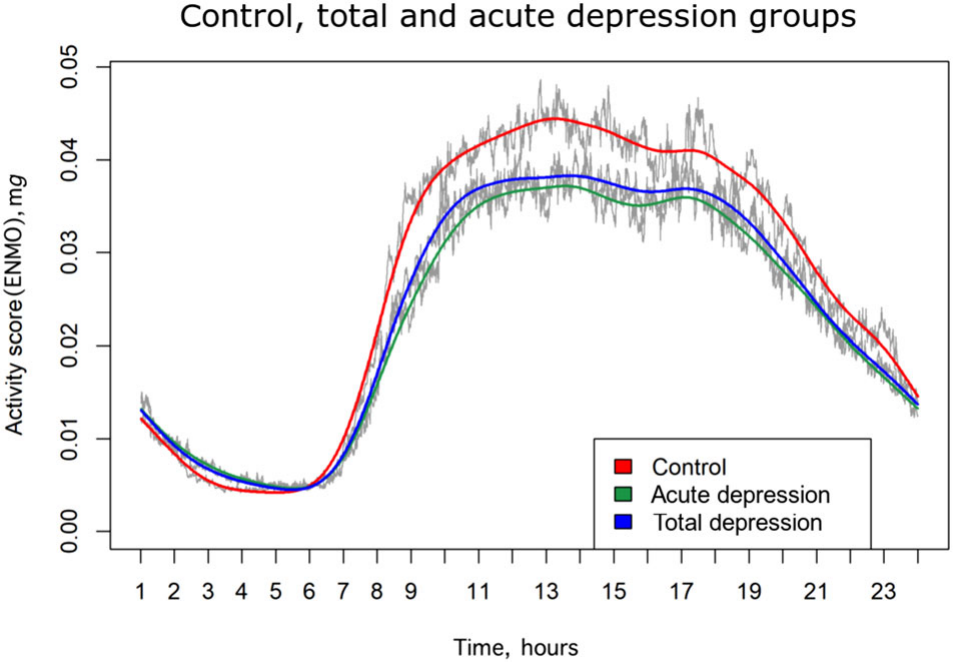
Twenty-four-hour averaged activity scores across 14 days within different diagnostic (sub-)groups. The acute depression group is the subgroup of the total depression group.

Having a diagnosis of depressive disorder in the past 6 months was significantly associated with lower MESOR in both the unadjusted (Model 1) and adjusted model (Model 2). For amplitude, group status was significant in the unadjusted model (Model 1) and was associated with a lower amplitude which can be interpreted as a dampened rhythm, but not in the adjusted model (Table 3, Model 2, *p* = 0.065). Group status was not significantly associated with acrophase expressed in any model, but there was a significant relationship between chronotype and acrophase with later chronotype having later acrophase (Model 3). To ease interpretation of the statistical results, averaged 24-h activity rhythms are visualized per group in Fig. 1.

**Table 3:** Associations between physical activity parameters and depression status (total depression (past 6 months) vs. controls).

Characteristic	Model 1				Model 2				Model 3			
	*B*	SE *B*	*p*	95%CI	*B*	SE *B*	*p*	95%CI	*B*	SE *B*	*p*	95%CI
MESOR												
Fixed effects												
Intercept	0.027	0.001	**<0.001**	0.025; 0.029	0.038	0.005	**<0.001**	0.028; 0.048				
Group status^a^	−0.003	0.001	**0.008**	−0.005; −0.001	−0.003	0.001	**0.033**	−0.005; −0.001				
Random effects												
Intercept	3.806^−5^	5.315^−6^	**<0.001**	2.895^−5^; 5.005^−5^	3.225^−5^	4.726^−6^	**<0.001**	2.420^−5^; 4.298^−5^				
Amplitude												
Fixed effects												
Intercept	0.022	0.001	**<0.001**	0.020; 0.023	0.031	0.005	**<0.001**	0.021; 0.041				
Group status	−0.002	0.001	**0.039**	−0.005; −0.001	−0.002	0.001	0.065	−0.005; 0.001				
Random effects												
Intercept	3.545^−5^	5.293^−6^	**<0.001**	2.646^−5^; 4.751^−5^	2.961^−5^	4.703^−6^	**<0.001**	2.169^−5^; 4.043^−5^				
Acrophase												
Fixed effects												
Intercept	14.602	0.129	**<0.001**	14.345; 14.859	15.482	0.912	**<0.001**	13.674; 17.289	12.851	0.321	**<0.001**	12.213; 13.488
Group status	0.156	0.187	0.407	−0.215; 0.527	0.206	0.243	0.398	−0.275; 0.689	0.063	0.157	0.678	−0.248; 0.375
Chronotype									0.457	0.077	**<0.001**	0.0304; 0.610
Random effects												
Intercept	0.873	0.138	**<0.001**	0.640; 1.191	0.920	0.149	**<0.001**	0.669; 1.265	0.497	0.094	**<0.001**	0.343; 0.721

Model 1 is a baseline model without covariates. Model 2 is adjusted for age, gender, marital status, employment, education, BMI, smoking status, medication use (antidepressants use, benzodiazepines use, antipsychotics use, and lithium use). Model 3 is additionally adjusted for chronotype.

^a^The reference is the control group.

*P*-values at or below 0.05 are marked in bold.

### Actigraphy differences between acutely depressed participants (1-month cut-off) and controls

Table 4 shows the results of the repeated analyses in a subsample of depressed individuals who were more likely to suffer from acute depressive symptoms. Acute depressive disorders were significantly associated with decreased MESOR in both unadjusted and adjusted models. Acute depressive disorders were associated with significantly lower amplitude in both unadjusted and adjusted models, which indicates the dampened rhythm in the depressed group. Acute depressive disorders were not significantly associated with acrophase in any model, but there was a marginally significant effect towards the later timing of activity in the adjusted model. There was a significant relationship between chronotype and acrophase with later chronotype having later acrophase. The subgroup analysis in this more homogeneous depressed group revealed overall more pronounced results.

**Table 4:** Associations between physical activity parameters and depression status (acute depression (past 1 month) vs. controls).

Characteristic	Model 1				Model 2				Model 3			
	*B*	SE *B*	*p*	95%CI	*B*	SE *B*	*p*	95%CI	*B*	SE *B*	*p*	95%CI
MESOR												
Fixed effects												
Intercept	0.027	0.001	**<0.001**	0.025; 0.028	0.038	0.005	**<0.001**	0.028; 0.048				
Group status^a^	−0.004	0.001	**0.002**	−0.006; −0.001	−0.004	0.001	**0.005**	−0.006; −0.001				
Random effects												
Intercept	3.705^−5^	5.184^−6^	**<0.001**	2.817^−5^; 4.875^−5^	3.123^−5^	4.589^−6^	**<0.001**	2.341^−5^; 4.165^−5^				
Amplitude												
Fixed effects												
Intercept	0.022	0.001	**<0.001**	0.020; 0.023	0.031	0.005	**<0.001**	0.021; 0.041				
Group status^a^	−0.003	0.001	**0.009**	−0.005; −0.001	−0.003	0.001	**0.011**	−0.005; −0.001				
Random effects												
Intercept	3.459^−5^	5.181^−6^	**<0.001**	2.579^−5^; 4.640^−5^	2.866^−5^	4.574^−6^	**<0.001**	2.096^−5^; 3.918^−5^				
Acrophase												
Fixed effects												
Intercept	14.575	0.116	**<0.001**	14.345; 14.805	15.461	0.899	**<0.001**	13.679; 17.244	12.813	0.318	**<0.001**	12.182; 13.444
Group status^a^	0.286	0.194	0.143	−0.098; 0.671	0.405	0.232	0.084	−0.055; 0.866	0.188	0.162	0.248	−0.133; 0.510
Chronotype									0.456	0.077	**<0.001**	0.304; 0.608
Random effects												
Intercept	0.860	0.136	**<0.001**	0.630; 1.174	0.898	0.146	**<0.001**	0.652; 1.236	0.490	0.093	**<0.001**	0.337; 0.711

Model 1 is an unadjusted model without covariates. Model 2 is adjusted for age, gender, marital status, employment, education, BMI, smoking status, medication use (antidepressants use, benzodiazepines use, antipsychotics use, and lithium use). Model 3 is additionally adjusted for chronotype.

^a^Depressed group was defined as having a depression diagnosis 1 month before the assessment; the reference is the control group.

*P*-values at or below 0.05 are marked in bold.

## Discussion

We assessed objective actigraphy-measured level and timing of PA in depressed individuals and controls. Supporting the first hypothesis, MESOR, the rhythm-corrected mean level, was lower in depressed individuals, compared to controls. This result was confirmed in both total depression (6-month) and in the subset of acute depression (1-month) group. The amplitude, as a measure of the difference between activity during the day and the night, was also lower in the subgroup of acutely depressed individuals compared to controls, but not in the total depression group. Our hypothesis that depressed individuals showed a later timing of PA during their normal daily life was not confirmed, notwithstanding the marginally significant association towards the later timing in the acute depression group.

The finding that average PA levels were significantly reduced in depressed individuals compared to the control group is consistent with findings from other actigraphy studies^13–15,18^, which used slightly different approaches for estimating average 24-h activity levels. Interestingly, for the level, we do find effects in both the total and the acute depression groups, suggesting that lower levels of activity may also be present during a recovery phase of a depressive episode. Lower activity could, therefore, be interpreted as a relative persistent vulnerability marker for depression and may even be present before developing depression or relapsing into a next episode. This interpretation, however, is speculative and needs to be confirmed in future prospective research.

Another important finding with respect to the diurnal rhythm of activity is that dampened amplitude was found in the acute depression group. Although these results could either mean that depressed individuals reach lower activity levels at daytime or that they have more nighttime activity than controls, activity pattern (Fig. 1) illustrates that the difference in amplitude results from the first possibility. As the association was only statistically significant among acutely depressed individuals compared to controls, but not in the larger group of individuals who were not all necessarily in an active depressive episode (total depression group), this suggests that dampened amplitude is a state effect of depression. A previous NESDA study showed that the median duration of a depressive episode is 6 months in the sample^51^. Therefore, it is possible that some individuals who scored positive on the 6-month threshold had already recovered at the moment of the actigraphy measures. This is supported by the reported IDS scores among the total depression group, which indicates that a third of the subjects had no (13.8%) or mild depressive symptoms (19%). Hence, the finding may reflect that the amplitude normalizes and becomes less dampened after individuals recover from their acute depressive episode. Indeed, in line with our findings, studies comparing depressed individuals before and after treatment showed an improvement in intensity and increase in amplitude of daytime activity^15^. In future studies, it may be interesting to explore whether changes in the amplitude do not only go together with a change in depression status but perhaps even precede it. In that case, it could be used as an early-warning signal for onset and recovery.

We found a marginally significant effect towards later timing to be associated with acute depression only in the adjusted model. This nonsignificant finding might be a reflection of an insufficient number of acutely depressed individuals for detecting a relatively small difference in the timing of activity. Additionally, even in the acute depression group, we cannot be sure that everyone was in a depressed state at the moment of assessment. The IDS scores (Table 1) support this idea, as there were some individuals with no (7%) and mild depressive symptoms (9.3%) in the acute depression group also. However, in two prior studies, similar marginally significant effects towards the later timing of activity in depressed individuals compared to controls were reported as well^13,33^. One study had a considerably small sample size (*n* = 40)^13^, and another one used narrow population groups (elderly females); therefore, the findings may not be applicable to other populations^33^. When studying the sleep-wake and activity cycles among young adults with various emerging mental disorders, depressed individuals showed a significantly later acrophase compared to controls^52^. This might be explained by studying much younger participants (20.0 ± 4.4 years) accompanied by greater medication use and comorbidities (that presume more severe depressive symptoms) and/or more eveningness. Similarly, a later peak of 24-h activity was observed in young (≤39 years, 45 min of delay) compared to older participants (≥40 years, 30 min of delay), and in those with more depressive symptoms^53^.

Our study has strengths and limitations. Among the strengths is the large sample size including individuals with clinically diagnosed depressive disorder and individuals without a lifetime history of depression. Moreover, we used the cosinor method, which is one of the most commonly used reliable methods, which allowed us to estimate different characteristics of the level (MESOR and amplitude) and occurrence of the activity peak (acrophase)^41,42^. A limitation of using the cosinor analysis is that it is a parametric analysis, which fits a symmetric cosinor curve into the circadian rhythm. However, the circadian rhythm of daily activity is not symmetric, with a shorter “night-curve” and a longer “day-curve”. Moreover, it is known that based on the cosinor method, it is hard to undoubtfully detect when the physically active period starts and how long it lasts. Other nonparametric approaches are available now, but most of them aggregate data across all available days and not day-by-day^18^, reducing power. To overcome this limitation in future studies, more advanced analytical tools have to be used that can improve our understanding of the circadian rest-activity rhythm. We also excluded a part of the sample, the remitted depressed individuals, what consequently reduces the sample size. The main reason was that the aim of this study was not originally to include the remitted individuals, as we wanted to compare current cases versus controls. Additionally, it has been recently shown that remitted depressed individuals did not differ from non-depressed in their diurnal rest-activity rhythm^18^. Another limitation of this study is that a small number of individuals in the depression groups reported no or mild depressive symptoms. This was most likely due to logistic reasons typical for large cohort studies as our study is. Participants were diagnosed with the CIDI instrument during the regular NESDA interview wave, which was maximal 31 days prior to the actigraphy assessments, whereas depression severity assessment with the IDS questionnaire was not necessarily done close to the actigraphy assessment period (up to 72 days prior and two cases of 74 and 351 days after the CIDI). Finally, the cross-sectional nature of the study does not allow drawing conclusions regarding the direction of the association between the diagnosis of depression and PA.

We would recommend for future actigraphy studies in depressed patients to not only study the level and the timing of PA but also rhythm characteristics and their combinations. Eight subgroups with various rhythm characteristics in terms of level, robustness, length, and timing were identified in elderly men^14^. Smagula et al. concluded that different combinations of those characteristics have different associations with depressive symptoms. It might be valuable to use a combination of the cosinor and nonparametric analyses, that will allow taking into account both parametric and nonparametric rhythm-related parameters. Finally, longitudinal studies are needed to disentangle the direction of the association between depression and daily rest-activity rhythms. This knowledge may help to determine a pathophysiologic pathway leading to depressive disorders and to develop better treatments.

In conclusion, our study showed that the level of activity, specifically during the daytime, was significantly lower in individuals in both the total and the acute depression groups compared to controls. Significantly lower day/night differences were found only in the acute depression group. The later timing of activity was only marginally significantly associated with acute depression. These findings not only indicate an important role of the level of PA across both depression groups but also the additional changes in the robustness of the diurnal rhythm (more dampened amplitude of the rhythm), which are associated with the more acute depression state. Hence, our study provided new relevant insights on how different aspects of physical activity, namely level and timing, may be independently associated to depression. Future studies should examine the timing of activity in a larger sample with acutely depressed individuals to be able to draw more firm conclusions.

## Supplementary information

Supplementary Material
