# Supplementary material for: Level and timing of physical activity during normal daily life in depressed and non-depressed individuals
Source: Transl Psychiatry. 2020 Jul 30;10:259. doi: 10.1038/s41398-020-00952-w (PMC7393081; doi:10.1038/s41398-020-00952-w)
Supplement: Supplementary file 1 — Supplementary Material [file 41398_2020_952_MOESM1_ESM.docx]

## Supplementary Material 1

**R script for a cosinor analysis with daily values**

**library**(tidyverse)
**library**(lubridate)
**library**(dplyr)
**library**(cosinor)
**library**(cosinor2)

get_id <- **function**(filename) {
 pts <- **as.vector**(stringr**::str_split**(filename, "_", simplify = TRUE))
 **return**(**paste0**(pts[1], "__", pts[3]))
}
read_data <- **function**(filename, dir = data_dir) {
 path <- **paste0**(data_dir, filename)
 data <- **read_csv**(path)

 **if**(**format**(data**$**timestamp[1], '%H') **==** '17') {
 data <- **remove_first_last_days**(data, starts_at = 17)
 data**$**timestamp <- data**$**timestamp **+** 2*****60*****60
 } **else** {
 data <- **remove_first_last_days**(data, starts_at = 18)
 data**$**timestamp <- data**$**timestamp **+** 60*****60
 }
 data**$**id <- **get_id**(filename)
 data**$**hour <- **hour**(data**$**timestamp)
 data**$**day <- **day**(data**$**timestamp)
 data**$**month <- **month**(data**$**timestamp)
 data**$**year <- **year**(data**$**timestamp)
 **return**(data)
}

remove_first_last_days <- **function**(data_file, starts_at) {
 **if**(starts_at **==** 17) {
 *# delete all observations of first day up until 22:00,*
 *# the last day, as well as the last two hours of the day before the last*
 upper_bound <- **paste0**(data_file**$**timestamp[1] **%>%** **format**('%Y-%m-%d'), ' ', '22:00:00') **%>%** ymd_hms
 first_part_ind <- **which**(data_file**$**timestamp **<** upper_bound)
 second_to_last_day <- (**ymd_hms**(data_file**$**timestamp[**length**(data_file**$**timestamp)]) **-** **days**(1)) **%>%** **format**('%Y-%m-%d')
 lower_bound <- **paste0**(second_to_last_day, ' ', '22:00:00') **%>%** ymd_hms
 last_part_ind <- **which**(lower_bound **<=** data_file**$**timestamp)
 new_data <- data_file[**-c**(first_part_ind, last_part_ind), ]
 **return**(new_data)
 } **else** {
 *# delete all observations of first day up until 23:00,*
 *# the last day, as well as the last hour of the day before the last*
 upper_bound <- **paste0**(data_file**$**timestamp[1] **%>%** **format**('%Y-%m-%d'), ' ', '23:00:00') **%>%** ymd_hms
 first_part_ind <- **which**(data_file**$**timestamp **<** upper_bound)

 second_to_last_day <- (**ymd_hms**(data_file**$**timestamp[**length**(data_file**$**timestamp)]) **-** **days**(1)) **%>%** **format**('%Y-%m-%d')
 lower_bound <- **paste0**(second_to_last_day, ' ', '23:00:00') **%>%** ymd_hms
 last_part_ind <- **which**(lower_bound **<=** data_file**$**timestamp)
 new_data <- data_file[**-c**(first_part_ind, last_part_ind), ]
 **return**(new_data)
 }
}
fit_cosinor <- **function**(df, output_coefs = TRUE) {
 *# If you want to change the model-fitting part, change this function*
 m <- **cosinor.lm**(ENMO **~** **time**(time), data = **as.data.frame**(df), period = 24)
 m_coefs <- **c**(m**$**coefficients)
 corrected_acrophase <- **correct.acrophase**(m)
 m_coefs[3] <- corrected_acrophase
 coef_names <- **names**(m_coefs)
 m_coefs[4] <- **abs**(m_coefs[3])
 **names**(m_coefs) <- **c**(coef_names, 'abs_acr')
 **return**(m_coefs)
}

process_data_files <- **function**(data_file_name, coefs_only = TRUE) {
 data_file <- **read_data**(data_file_name)
 timestamps <- data_file **%>%** **group_by**(day) **%>%** **summarise**(timestamp = **head**(**as.character**(timestamp), 1))
 coefficients_per_day <- data_file **%>%**
 **mutate**(hour = **hour**(timestamp)) **%>%**
 **group_by**(month, day) **%>%**
 **mutate**(time = 1**:n**()**/**60) **%>%**
 **ungroup**(month, day) **%>%**
 as_tibble **%>%**
 **split**(.**$**day, drop = TRUE) **%>%**
 **sapply**(fit_cosinor, output_coefs = TRUE) **%>%**
 t **%>%**
 as_tibble

 coefficients_per_day**$**timestamp <- timestamps**$**timestamp
 coefficients_per_day**$**id <- **get_id**(data_file_name)
 coefficients_per_day**$**group <- ID_dataframe[**which**(ID_dataframe **==** coefficients_per_day**$**id, arr.ind = T)[1], 2] **%>%** as.numeric
 **return**(coefficients_per_day)
}

control_ids <- **list.files**('Data/controls/') **%>%** **sapply**(get_id) *# change the directory path as needed*
depressed_ids <- **list.files**('Data/depressed/') **%>%** **sapply**(get_id) *# change the directory path as needed*
ID_dataframe <- **as_tibble**(**list**(ID = **c**(control_ids, depressed_ids), group = **c**(**rep**(0, 63), **rep**(1, 58))))

data_dir <- 'Data/combined/' *# change the directory path as needed*
data_file_names <- data_dir **%>%** **list.files**(pattern = '*.csv')

coefficients_per_person <- **lapply**(data_file_names, process_data_files) **%>%** **lapply**(**function**(df) **return**(df **%>%** **arrange**(timestamp)))
models_per_person <- **lapply**(data_file_names, process_data_files, coefs_only = FALSE)

*# This is the final result:*
out <- **do.call**('rbind', coefficients_per_person)

*# Writing the final result to file:*
**write_csv**(out, 'coefficients_per_day.csv')

## Supplementary Material 2

**The syntax and the results of the bootstrap analyses**

* Encoding: UTF-8.

*Create lagged values.

SHIFT VALUES VARIABLE=Intercept RESULT=lag1intercept LAG=1.

execute.

SHIFT VALUES VARIABLE=amp RESULT=lag1amp LAG=1.

execute.

SHIFT VALUES VARIABLE=acr_hours RESULT=lag1acr_hours LAG=1.

execute.

*********************************************************

****MODELS for the total depression group****

*********************************************************

*MODEL 1. Intercept (MESOR) with lagged fixed and random effect instead of repeated statement.

MIXED Intercept WITH group lag1intercept

/CRITERIA=CIN(95) MXITER(100) MXSTEP(10) SCORING(1) SINGULAR(0.000000000001) HCONVERGE(0,

ABSOLUTE) LCONVERGE(0, ABSOLUTE) PCONVERGE(0.000001, ABSOLUTE)

/FIXED=group lag1intercept | SSTYPE(3)

/METHOD=REML

/PRINT=SOLUTION TESTCOV

/RANDOM=INTERCEPT lag1intercept | SUBJECT(pident) COVTYPE(VC)

/SAVE=PRED RESID.

*Bootstrap. MESOR.

PRESERVE.

SET RNG=MT MTINDEX=5.

SHOW RNG.

BOOTSTRAP

/SAMPLING METHOD=SIMPLE

/VARIABLES TARGET=Intercept INPUT= group lag1intercept

/CRITERIA CILEVEL=95 CITYPE=PERCENTILE NSAMPLES=1000

/MISSING USERMISSING=EXCLUDE.

MIXED Intercept WITH group lag1intercept

/CRITERIA=CIN(95) MXITER(100) MXSTEP(10) SCORING(1) SINGULAR(0.000000000001) HCONVERGE(0,

ABSOLUTE) LCONVERGE(0, ABSOLUTE) PCONVERGE(0.000001, ABSOLUTE)

/FIXED=group lag1intercept | SSTYPE(3)

/METHOD=REML

/PRINT=SOLUTION TESTCOV

/RANDOM=INTERCEPT lag1intercept | SUBJECT(pident) COVTYPE(VC).

RESTORE.

*MODEL 2. Intercept (MESOR) with lagged fixed and random effect instead of repeated statement + all covariates.

MIXED Intercept WITH group lag1intercept fage sex fedu fmarstat femplst fsmoke1 fbmi psychmed

/CRITERIA=CIN(95) MXITER(100) MXSTEP(10) SCORING(1) SINGULAR(0.000000000001) HCONVERGE(0,

ABSOLUTE) LCONVERGE(0, ABSOLUTE) PCONVERGE(0.000001, ABSOLUTE)

/FIXED=group lag1intercept fage sex fedu fmarstat femplst fsmoke1 fbmi psychmed| SSTYPE(3)

/METHOD=REML

/PRINT=SOLUTION TESTCOV

/RANDOM=INTERCEPT lag1intercept | SUBJECT(pident) COVTYPE(VC)

/SAVE=PRED RESID.

*Bootstrap. MESOR.

PRESERVE.

SET RNG=MT MTINDEX=5.

SHOW RNG.

BOOTSTRAP

/SAMPLING METHOD=SIMPLE

/VARIABLES TARGET=Intercept INPUT= group lag1intercept

/CRITERIA CILEVEL=95 CITYPE=PERCENTILE NSAMPLES=1000

/MISSING USERMISSING=EXCLUDE.

MIXED Intercept WITH group lag1intercept fage sex fedu fmarstat femplst fsmoke1 fbmi psychmed

/CRITERIA=CIN(95) MXITER(100) MXSTEP(10) SCORING(1) SINGULAR(0.000000000001) HCONVERGE(0,

ABSOLUTE) LCONVERGE(0, ABSOLUTE) PCONVERGE(0.000001, ABSOLUTE)

/FIXED=group lag1intercept fage sex fedu fmarstat femplst fsmoke1 fbmi psychmed| SSTYPE(3)

/METHOD=REML

/PRINT=SOLUTION TESTCOV

/RANDOM=INTERCEPT lag1intercept | SUBJECT(pident) COVTYPE(VC).

RESTORE.

*MODEL 1. Amplitude with lagged fixed and random effect instead of repeated statement.

MIXED amp WITH group lag1amp

/CRITERIA=CIN(95) MXITER(100) MXSTEP(10) SCORING(1) SINGULAR(0.000000000001) HCONVERGE(0,

ABSOLUTE) LCONVERGE(0, ABSOLUTE) PCONVERGE(0.000001, ABSOLUTE)

/FIXED=group lag1amp | SSTYPE(3)

/METHOD=REML

/PRINT=SOLUTION TESTCOV

/RANDOM=INTERCEPT lag1amp | SUBJECT(pident) COVTYPE(VC)

/SAVE=PRED RESID.

*Bootstrap. ampl.

PRESERVE.

SET RNG=MT MTINDEX=5.

SHOW RNG.

BOOTSTRAP

/SAMPLING METHOD=SIMPLE

/VARIABLES TARGET=amp INPUT= group lag1amp

/CRITERIA CILEVEL=95 CITYPE=PERCENTILE NSAMPLES=1000

/MISSING USERMISSING=EXCLUDE.

MIXED amp WITH group lag1amp

/CRITERIA=CIN(95) MXITER(100) MXSTEP(10) SCORING(1) SINGULAR(0.000000000001) HCONVERGE(0,

ABSOLUTE) LCONVERGE(0, ABSOLUTE) PCONVERGE(0.000001, ABSOLUTE)

/FIXED=group lag1amp | SSTYPE(3)

/METHOD=REML

/PRINT=SOLUTION TESTCOV

/RANDOM=INTERCEPT lag1amp | SUBJECT(pident) COVTYPE(VC).

RESTORE.

*MODEL 2. Amplitude with lagged fixed and random effect instead of repeated statement + all covariates.

MIXED amp WITH group lag1amp fage sex fedu fmarstat femplst fsmoke1 fbmi psychmed

/CRITERIA=CIN(95) MXITER(100) MXSTEP(10) SCORING(1) SINGULAR(0.000000000001) HCONVERGE(0,

ABSOLUTE) LCONVERGE(0, ABSOLUTE) PCONVERGE(0.000001, ABSOLUTE)

/FIXED=group lag1amp fage sex fedu fmarstat femplst fsmoke1 fbmi psychmed | SSTYPE(3)

/METHOD=REML

/PRINT=SOLUTION TESTCOV

/RANDOM=INTERCEPT lag1amp | SUBJECT(pident) COVTYPE(VC)

/SAVE=PRED RESID.

*Bootstrap. amplitude

PRESERVE.

SET RNG=MT MTINDEX=5.

SHOW RNG.

BOOTSTRAP

/SAMPLING METHOD=SIMPLE

/VARIABLES TARGET=amp INPUT= group lag1amp

/CRITERIA CILEVEL=95 CITYPE=PERCENTILE NSAMPLES=1000

/MISSING USERMISSING=EXCLUDE.

MIXED amp WITH group lag1amp fage sex fedu fmarstat femplst fsmoke1 fbmi psychmed

/CRITERIA=CIN(95) MXITER(100) MXSTEP(10) SCORING(1) SINGULAR(0.000000000001) HCONVERGE(0,

ABSOLUTE) LCONVERGE(0, ABSOLUTE) PCONVERGE(0.000001, ABSOLUTE)

/FIXED=group lag1amp fage sex fedu fmarstat femplst fsmoke1 fbmi psychmed| SSTYPE(3)

/METHOD=REML

/PRINT=SOLUTION TESTCOV

/RANDOM=INTERCEPT lag1amp | SUBJECT(pident) COVTYPE(VC).

RESTORE.

*MODEL 1. Acrophase with lagged fixed and random effect instead of repeated statement.

MIXED acr_hours WITH group lag1acr_hours

/CRITERIA=CIN(95) MXITER(100) MXSTEP(10) SCORING(1) SINGULAR(0.000000000001) HCONVERGE(0,

ABSOLUTE) LCONVERGE(0, ABSOLUTE) PCONVERGE(0.000001, ABSOLUTE)

/FIXED=group lag1acr_hours | SSTYPE(3)

/METHOD=REML

/PRINT=SOLUTION TESTCOV

/RANDOM=INTERCEPT lag1acr_hours | SUBJECT(pident) COVTYPE(VC)

/SAVE=PRED RESID.

*Bootstrap. acrophase.

PRESERVE.

SET RNG=MT MTINDEX=5.

SHOW RNG.

BOOTSTRAP

/SAMPLING METHOD=SIMPLE

/VARIABLES TARGET=acr_hours INPUT= group lag1acr_hours

/CRITERIA CILEVEL=95 CITYPE=PERCENTILE NSAMPLES=1000

/MISSING USERMISSING=EXCLUDE.

MIXED acr_hours WITH group lag1acr_hours

/CRITERIA=CIN(95) MXITER(100) MXSTEP(10) SCORING(1) SINGULAR(0.000000000001) HCONVERGE(0,

ABSOLUTE) LCONVERGE(0, ABSOLUTE) PCONVERGE(0.000001, ABSOLUTE)

/FIXED=group lag1acr_hours | SSTYPE(3)

/METHOD=REML

/PRINT=SOLUTION TESTCOV

/RANDOM=INTERCEPT lag1acr_hours | SUBJECT(pident) COVTYPE(VC).

RESTORE.

*MODEL 2. Acrophase with lagged fixed and random effect instead of repeated statement + all covariates.

MIXED acr_hours WITH group lag1acr_hours fage sex fedu fmarstat femplst fsmoke1 fbmi psychmed

/CRITERIA=CIN(95) MXITER(100) MXSTEP(10) SCORING(1) SINGULAR(0.000000000001) HCONVERGE(0,

ABSOLUTE) LCONVERGE(0, ABSOLUTE) PCONVERGE(0.000001, ABSOLUTE)

/FIXED=group lag1acr_hours fage sex fedu fmarstat femplst fsmoke1 fbmi psychmed| SSTYPE(3)

/METHOD=REML

/PRINT=SOLUTION TESTCOV

/RANDOM=INTERCEPT lag1acr_hours | SUBJECT(pident) COVTYPE(VC)

/SAVE=PRED RESID.

*Bootstrap. acrophase.

PRESERVE.

SET RNG=MT MTINDEX=5.

SHOW RNG.

BOOTSTRAP

/SAMPLING METHOD=SIMPLE

/VARIABLES TARGET=acr_hours INPUT= group lag1acr_hours

/CRITERIA CILEVEL=95 CITYPE=PERCENTILE NSAMPLES=1000

/MISSING USERMISSING=EXCLUDE.

MIXED acr_hours WITH group lag1acr_hours fage sex fedu fmarstat femplst fsmoke1 fbmi psychmed

/CRITERIA=CIN(95) MXITER(100) MXSTEP(10) SCORING(1) SINGULAR(0.000000000001) HCONVERGE(0,

ABSOLUTE) LCONVERGE(0, ABSOLUTE) PCONVERGE(0.000001, ABSOLUTE)

/FIXED=group lag1acr_hours fage sex fedu fmarstat femplst fsmoke1 fbmi psychmed | SSTYPE(3)

/METHOD=REML

/PRINT=SOLUTION TESTCOV

/RANDOM=INTERCEPT lag1acr_hours | SUBJECT(pident) COVTYPE(VC).

RESTORE.

* MODEL 3. Acrophase with lagged fixed effects and random effect instead of repeated statement + chronotype.

MIXED acr_hours WITH group MSFsc

/CRITERIA=CIN(95) MXITER(100) MXSTEP(10) SCORING(1) SINGULAR(0.000000000001) HCONVERGE(0,

ABSOLUTE) LCONVERGE(0, ABSOLUTE) PCONVERGE(0.000001, ABSOLUTE)

/FIXED=group MSFsc | SSTYPE(3)

/METHOD=REML

/PRINT=SOLUTION TESTCOV

/RANDOM=INTERCEPT | SUBJECT(pident) COVTYPE(VC)

/SAVE=PRED RESID.

*Bootstrap. acrophase.

PRESERVE.

SET RNG=MT MTINDEX=5.

SHOW RNG.

BOOTSTRAP

/SAMPLING METHOD=SIMPLE

/VARIABLES TARGET=acr_hours INPUT= group lag1acr_hours

/CRITERIA CILEVEL=95 CITYPE=PERCENTILE NSAMPLES=1000

/MISSING USERMISSING=EXCLUDE.

MIXED acr_hours WITH group MSFsc

/CRITERIA=CIN(95) MXITER(100) MXSTEP(10) SCORING(1) SINGULAR(0.000000000001) HCONVERGE(0,

ABSOLUTE) LCONVERGE(0, ABSOLUTE) PCONVERGE(0.000001, ABSOLUTE)

/FIXED= group MSFsc | SSTYPE(3)

/METHOD=REML

/PRINT=SOLUTION TESTCOV

/RANDOM=INTERCEPT lag1acr_hours | SUBJECT(pident) COVTYPE(VC).

RESTORE.

**********************************************************

****MODELS for the acute depression group****

**********************************************************

* Selecting only currently depressed and control cases.

USE ALL.

COMPUTE filter_$=(monthgroup < 2).

VARIABLE LABELS filter_$ 'monthgroup < 2 (FILTER)'.

VALUE LABELS filter_$ 0 'Not Selected' 1 'Selected'.

FORMATS filter_$ (f1.0).

FILTER BY filter_$.

EXECUTE.

*MODEL 1. Intercept (MESOR) with lagged fixed and random effect instead of repeated statement.

MIXED Intercept WITH monthgroup lag1intercept

/CRITERIA=CIN(95) MXITER(100) MXSTEP(10) SCORING(1) SINGULAR(0.000000000001) HCONVERGE(0,

ABSOLUTE) LCONVERGE(0, ABSOLUTE) PCONVERGE(0.000001, ABSOLUTE)

/FIXED=monthgroup lag1intercept | SSTYPE(3)

/METHOD=REML

/PRINT=SOLUTION TESTCOV

/RANDOM=INTERCEPT lag1intercept | SUBJECT(pident) COVTYPE(VC)

/SAVE=PRED RESID.

*Bootstrap. MESOR.

PRESERVE.

SET RNG=MT MTINDEX=5.

SHOW RNG.

BOOTSTRAP

/SAMPLING METHOD=SIMPLE

/VARIABLES TARGET=Intercept INPUT= monthgroup lag1intercept

/CRITERIA CILEVEL=95 CITYPE=PERCENTILE NSAMPLES=1000

/MISSING USERMISSING=EXCLUDE.

MIXED Intercept WITH monthgroup lag1intercept

/CRITERIA=CIN(95) MXITER(100) MXSTEP(10) SCORING(1) SINGULAR(0.000000000001) HCONVERGE(0,

ABSOLUTE) LCONVERGE(0, ABSOLUTE) PCONVERGE(0.000001, ABSOLUTE)

/FIXED=monthgroup lag1intercept | SSTYPE(3)

/METHOD=REML

/PRINT=SOLUTION TESTCOV

/RANDOM=INTERCEPT lag1intercept | SUBJECT(pident) COVTYPE(VC).

RESTORE.

*MODEL 2. Intercept (MESOR) with lagged fixed and random effect instead of repeated statement + all covariates.

MIXED Intercept WITH monthgroup lag1intercept fage sex fedu fmarstat femplst fsmoke1 fbmi psychmed

/CRITERIA=CIN(95) MXITER(100) MXSTEP(10) SCORING(1) SINGULAR(0.000000000001) HCONVERGE(0,

ABSOLUTE) LCONVERGE(0, ABSOLUTE) PCONVERGE(0.000001, ABSOLUTE)

/FIXED=monthgroup lag1intercept fage sex fedu fmarstat femplst fsmoke1 fbmi psychmed| SSTYPE(3)

/METHOD=REML

/PRINT=SOLUTION TESTCOV

/RANDOM=INTERCEPT lag1intercept | SUBJECT(pident) COVTYPE(VC)

/SAVE=PRED RESID.

*Bootstrap. MESOR.

PRESERVE.

SET RNG=MT MTINDEX=5.

SHOW RNG.

BOOTSTRAP

/SAMPLING METHOD=SIMPLE

/VARIABLES TARGET=Intercept INPUT= monthgroup lag1intercept

/CRITERIA CILEVEL=95 CITYPE=PERCENTILE NSAMPLES=1000

/MISSING USERMISSING=EXCLUDE.

MIXED Intercept WITH monthgroup lag1intercept fage sex fedu fmarstat femplst fsmoke1 fbmi psychmed

/CRITERIA=CIN(95) MXITER(100) MXSTEP(10) SCORING(1) SINGULAR(0.000000000001) HCONVERGE(0,

ABSOLUTE) LCONVERGE(0, ABSOLUTE) PCONVERGE(0.000001, ABSOLUTE)

/FIXED=monthgroup lag1intercept fage sex fedu fmarstat femplst fsmoke1 fbmi psychmed| SSTYPE(3)

/METHOD=REML

/PRINT=SOLUTION TESTCOV

/RANDOM=INTERCEPT lag1intercept | SUBJECT(pident) COVTYPE(VC).

RESTORE.

*MODEL 1. Amplitude with lagged fixed and random effect instead of repeated statement.

MIXED amp WITH monthgroup lag1amp

/CRITERIA=CIN(95) MXITER(100) MXSTEP(10) SCORING(1) SINGULAR(0.000000000001) HCONVERGE(0,

ABSOLUTE) LCONVERGE(0, ABSOLUTE) PCONVERGE(0.000001, ABSOLUTE)

/FIXED=monthgroup lag1amp | SSTYPE(3)

/METHOD=REML

/PRINT=SOLUTION TESTCOV

/RANDOM=INTERCEPT lag1amp | SUBJECT(pident) COVTYPE(VC)

/SAVE=PRED RESID.

*Bootstrap. ampl.

PRESERVE.

SET RNG=MT MTINDEX=5.

SHOW RNG.

BOOTSTRAP

/SAMPLING METHOD=SIMPLE

/VARIABLES TARGET=amp INPUT= monthgroup lag1amp

/CRITERIA CILEVEL=95 CITYPE=PERCENTILE NSAMPLES=1000

/MISSING USERMISSING=EXCLUDE.

MIXED amp WITH monthgroup lag1amp

/CRITERIA=CIN(95) MXITER(100) MXSTEP(10) SCORING(1) SINGULAR(0.000000000001) HCONVERGE(0,

ABSOLUTE) LCONVERGE(0, ABSOLUTE) PCONVERGE(0.000001, ABSOLUTE)

/FIXED=monthgroup lag1amp | SSTYPE(3)

/METHOD=REML

/PRINT=SOLUTION TESTCOV

/RANDOM=INTERCEPT lag1amp | SUBJECT(pident) COVTYPE(VC).

RESTORE.

*MODEL 2. Amplitude with lagged fixed and random effect instead of repeated statement + all covariates.

MIXED amp WITH monthgroupoup lag1amp fage sex fedu fmarstat femplst fsmoke1 fbmi psychmed

/CRITERIA=CIN(95) MXITER(100) MXSTEP(10) SCORING(1) SINGULAR(0.000000000001) HCONVERGE(0,

ABSOLUTE) LCONVERGE(0, ABSOLUTE) PCONVERGE(0.000001, ABSOLUTE)

/FIXED=monthgroup lag1amp fage sex fedu fmarstat femplst fsmoke1 fbmi psychmed | SSTYPE(3)

/METHOD=REML

/PRINT=SOLUTION TESTCOV

/RANDOM=INTERCEPT lag1amp | SUBJECT(pident) COVTYPE(VC)

/SAVE=PRED RESID.

*Bootstrap. amplitude

PRESERVE.

SET RNG=MT MTINDEX=5.

SHOW RNG.

BOOTSTRAP

/SAMPLING METHOD=SIMPLE

/VARIABLES TARGET=amp INPUT= monthgroup lag1amp

/CRITERIA CILEVEL=95 CITYPE=PERCENTILE NSAMPLES=1000

/MISSING USERMISSING=EXCLUDE.

MIXED amp WITH monthgroup lag1amp fage sex fedu fmarstat femplst fsmoke1 fbmi psychmed

/CRITERIA=CIN(95) MXITER(100) MXSTEP(10) SCORING(1) SINGULAR(0.000000000001) HCONVERGE(0,

ABSOLUTE) LCONVERGE(0, ABSOLUTE) PCONVERGE(0.000001, ABSOLUTE)

/FIXED=monthgroup lag1amp fage sex fedu fmarstat femplst fsmoke1 fbmi psychmed| SSTYPE(3)

/METHOD=REML

/PRINT=SOLUTION TESTCOV

/RANDOM=INTERCEPT lag1amp | SUBJECT(pident) COVTYPE(VC).

RESTORE.

*MODEL 1. Acrophase with lagged fixed and random effect instead of repeated statement.

MIXED acr_hours WITH monthgroup lag1acr_hours

/CRITERIA=CIN(95) MXITER(100) MXSTEP(10) SCORING(1) SINGULAR(0.000000000001) HCONVERGE(0,

ABSOLUTE) LCONVERGE(0, ABSOLUTE) PCONVERGE(0.000001, ABSOLUTE)

/FIXED=monthgroup lag1acr_hours | SSTYPE(3)

/METHOD=REML

/PRINT=SOLUTION TESTCOV

/RANDOM=INTERCEPT lag1acr_hours | SUBJECT(pident) COVTYPE(VC)

/SAVE=PRED RESID.

*Bootstrap. acrophase.

PRESERVE.

SET RNG=MT MTINDEX=5.

SHOW RNG.

BOOTSTRAP

/SAMPLING METHOD=SIMPLE

/VARIABLES TARGET=acr_hours INPUT= group lag1acr_hours

/CRITERIA CILEVEL=95 CITYPE=PERCENTILE NSAMPLES=1000

/MISSING USERMISSING=EXCLUDE.

MIXED acr_hours WITH monthgroup lag1acr_hours

/CRITERIA=CIN(95) MXITER(100) MXSTEP(10) SCORING(1) SINGULAR(0.000000000001) HCONVERGE(0,

ABSOLUTE) LCONVERGE(0, ABSOLUTE) PCONVERGE(0.000001, ABSOLUTE)

/FIXED=monthgroup lag1acr_hours | SSTYPE(3)

/METHOD=REML

/PRINT=SOLUTION TESTCOV

/RANDOM=INTERCEPT lag1acr_hours | SUBJECT(pident) COVTYPE(VC).

RESTORE.

*MODEL 2. Acrophase with lagged fixed and random effect instead of repeated statement + all covariates.

MIXED acr_hours WITH monthgroup lag1acr_hours fage sex fedu fmarstat femplst fsmoke1 fbmi psychmed

/CRITERIA=CIN(95) MXITER(100) MXSTEP(10) SCORING(1) SINGULAR(0.000000000001) HCONVERGE(0,

ABSOLUTE) LCONVERGE(0, ABSOLUTE) PCONVERGE(0.000001, ABSOLUTE)

/FIXED=monthgroup lag1acr_hours fage sex fedu fmarstat femplst fsmoke1 fbmi psychmed| SSTYPE(3)

/METHOD=REML

/PRINT=SOLUTION TESTCOV

/RANDOM=INTERCEPT lag1acr_hours | SUBJECT(pident) COVTYPE(VC)

/SAVE=PRED RESID.

*Bootstrap. acrophase.

PRESERVE.

SET RNG=MT MTINDEX=5.

SHOW RNG.

BOOTSTRAP

/SAMPLING METHOD=SIMPLE

/VARIABLES TARGET=acr_hours INPUT= group lag1acr_hours

/CRITERIA CILEVEL=95 CITYPE=PERCENTILE NSAMPLES=1000

/MISSING USERMISSING=EXCLUDE.

MIXED acr_hours WITH monthgroup lag1acr_hours fage sex fedu fmarstat femplst fsmoke1 fbmi psychmed

/CRITERIA=CIN(95) MXITER(100) MXSTEP(10) SCORING(1) SINGULAR(0.000000000001) HCONVERGE(0,

ABSOLUTE) LCONVERGE(0, ABSOLUTE) PCONVERGE(0.000001, ABSOLUTE)

/FIXED=monthgroup lag1acr_hours fage sex fedu fmarstat femplst fsmoke1 fbmi psychmed | SSTYPE(3)

/METHOD=REML

/PRINT=SOLUTION TESTCOV

/RANDOM=INTERCEPT lag1acr_hours | SUBJECT(pident) COVTYPE(VC).

RESTORE.

* MODEL 3. Acropase with lagged fixed effects and random effect instead of repeated statement + chronotype.

MIXED acr_hours WITH monthgroup MSFsc

/CRITERIA=CIN(95) MXITER(100) MXSTEP(10) SCORING(1) SINGULAR(0.000000000001) HCONVERGE(0,

ABSOLUTE) LCONVERGE(0, ABSOLUTE) PCONVERGE(0.000001, ABSOLUTE)

/FIXED=monthgroup MSFsc | SSTYPE(3)

/METHOD=REML

/PRINT=SOLUTION TESTCOV

/RANDOM=INTERCEPT | SUBJECT(pident) COVTYPE(VC)

/SAVE=PRED RESID.

*Bootstrap. acrophase.

PRESERVE.

SET RNG=MT MTINDEX=5.

SHOW RNG.

BOOTSTRAP

/SAMPLING METHOD=SIMPLE

/VARIABLES TARGET=acr_hours INPUT= group lag1acr_hours

/CRITERIA CILEVEL=95 CITYPE=PERCENTILE NSAMPLES=1000

/MISSING USERMISSING=EXCLUDE.

MIXED acr_hours WITH monthgroup MSFsc

/CRITERIA=CIN(95) MXITER(100) MXSTEP(10) SCORING(1) SINGULAR(0.000000000001) HCONVERGE(0,

ABSOLUTE) LCONVERGE(0, ABSOLUTE) PCONVERGE(0.000001, ABSOLUTE)

/FIXED= monthgroup MSFsc | SSTYPE(3)

/METHOD=REML

/PRINT=SOLUTION TESTCOV

/RANDOM=INTERCEPT lag1acr_hours | SUBJECT(pident) COVTYPE(VC).

RESTORE.

# Supplementary Material 3

**Table S1. Associations between physical activity parameters and group status (6-month cut-off) with bootstrap analyses**

| Characteristic | Model 1 | | | | Model 2 | | | | Model 3 | | | | |
| --- | --- | --- | --- | --- | --- | --- | --- | --- | --- | --- | --- | --- | --- |
|  | B | SE B | p | 95%CI | B | SE B | p | 95%CI | B | SE B | p | 95%CI |  |
| **MESOR** |  |  |  |  |  |  |  |  |  | | | |  |
| Fixed effects |  |  |  |  |  |  |  |  |  |  |  |  |  |
| Intercept | 0.025 | 0.001 | **0.001** | 0.024; 0.027 | 0.037 | 0.002 | **0.001** | 0.031; 0.039 |  |  |  |  |  |
| Group status | -0.003 | 0.001 | **0.001** | -0.004; -0.002 | -0.003 | 0.001 | **0.001** | -0.004; -0.002 |  |  |  |  |  |
| lag1intercept | 0.064 | 0.027 | **0.016** | -0.002; 0.101 | 0.062 | 0.027 | **0.016** | -0.005; 0.097 |  |  |  |  |  |
| Random effects |  |  |  |  |  |  |  |  |  |  |  |  |  |
| Intercept | 3.208^-5^ | 3.288^-6^ | **0.001** | 2.757^-5^; 3.489^-5^ | 2.809^-5^ | 3.018^-6^ | **0.001** | 2.759^-5^; 3.501^-5^ |  |  |  |  |  |
| lag1intercept | 0.003 | 0.005 | 0.979 | 0.006; 0.025 | 0.001 | 0.005 | 0.996 | 0.004; 0.023 |  |  |  |  |  |
| **Amplitude** |  |  |  |  |  |  |  |  |  | | | |  |
| Fixed effects |  |  |  |  |  |  |  |  |  |  |  |  |  |
| Intercept | 0.021 | 0.001 | **0.001** | 0.020; 0.023 | 0.030 | 0.002 | **0.001** | 0.026; 0.034 |  |  |  |  |  |
| Group status | -0.002 | 0.001 | **0.001** | -0.004; -0.002 | -0.003 | 0.001 | **0.001** | -0.005; 0.002 |  |  |  |  |  |
| lag1intercept | 0.040 | 0.027 | 0.174 | -0.033; 0.075 | 0.033 | 0.027 | 0.253 | -0.038; 0.068 |  |  |  |  |  |
| Random effects |  |  |  |  |  |  |  |  |  |  |  |  |  |
| Intercept | 2.963^-5^ | 4.651^-6^ | **0.001** | 2.897^-5^; 4.604^-5^ | 2.428^-5^ | 4.524^-6^ | **0.001** | 2.323^-5^; 4.034^-5^ |  |  |  |  |  |
| lag1intercept | 0.009 | 0.007 | 0.495 | 0.011; 0.042 | 0.010 | 0.008 | 0.329 | 0.011; 0.044 |  |  |  |  |  |
| **Acrophase** |  |  |  |  |  |  |  |  |  |  |  |  |  |
| Fixed effects |  |  |  |  |  |  |  |  |  |  |  |  |  |
| Intercept | 14.374 | 0.495 | **0.001** | 13.581; 15.523 | 15.479 | 0.668 | **0.001** | 14.244; 16.872 | 13.739 | 0.487 | **0.001** | 12.990; 14.890 |  |
| Group status | 0.151 | 0.086 | **0.046** | -0.015; 0.312 | 0.173 | 0.112 | 0.083 | -0.035; 0.402 | 0.145 | 0.086 | 0.053 | -0.017; 0.308 |  |
| lag1intercept | 0.014 | 0.034 | 0.725 | -0.064; 0.069 | 0.010 | 0.034 | 0.793 | -0.067; 0.066 | 0.013 | 0.035 | 0.747 | -0.066; 0.067 |  |
| Chronotype |  |  |  |  |  |  |  |  | 0.180 | 0.029 | **0.001** | 0.123; 0.239 |  |
| Random effects |  |  |  |  |  |  |  |  |  |  |  |  |  |
| Intercept | 0.457 | 0.212 | **0.001** | 0.054; 0.906 | 0.449 | 0.230 | **0.044** | 0.001; 0.905 | 0.401 | 0.216 | 0.064 | 0.026; 0.868 |  |
| lag1intercept | 0.002 | 0.001 | 0.274 | 0.001; 0.005 | 0.002 | 0.001 | 0.224 | 0.001; 0.006 | 0.002 | 0.01 | 0.274 | 0.001; 0.005 |  |

Note. Model 1 is a baseline model without covariates. Model 2 adjusted for age, gender, marital status, employment, education, BMI, smoking status, medication use (antidepressants use, benzodiazepines use, antipsychotics use, and lithium use). Model 3 adjusted for chronotype.

# Supplementary Material 4

**Table S2. Associations between physical activity parameters and group status (1-month cut-off) with bootstrap analyses**

| Characteristic | Model 1 | | | | Model 2 | | | | Model 3 | | | | |
| --- | --- | --- | --- | --- | --- | --- | --- | --- | --- | --- | --- | --- | --- |
|  | B | SE B | p | 95%CI | B | SE B | p | 95%CI | B | SE B | p | 95%CI |  |
| **MESOR** |  |  |  |  |  |  |  |  |  | | | |  |
| Fixed effects |  |  |  |  |  |  |  |  |  |  |  |  |  |
| Intercept | 0.025 | 0.001 | **0.001** | 0.024; 0.028 | 0.035 | 0.002 | **0.001** | 0.030; 0.039 |  |  |  |  |  |
| Group status* | -0.004 | 0.001 | **0.001** | -0.005; -0.003 | -0.004 | 0.001 | **0.001** | -0.006; -0.003 |  |  |  |  |  |
| lag1intercept | 0.058 | 0.029 | 0.052 | -0.013; 0.099 | 0.054 | 0.029 | 0.070 | -0.017; 0.096 |  |  |  |  |  |
| Random effects |  |  |  |  |  |  |  |  |  |  |  |  |  |
| Intercept | 2.939^-5^ | 3.766^-6^ | **0.001** | 2.546^-5^; 4.075^-5^ | 2.587^-5^ | 3.634^-6^ | **0.001** | 2.153^-5^; 3.613^-5^ |  |  |  |  |  |
| lag1intercept | 0.005 | 0.005 | 0.842 | 0.007; 0.026 | 0.003 | 0.005 | 0.915 | 0.006; 0.026 |  |  |  |  |  |
| **Amplitude** |  |  |  |  |  |  |  |  |  | | | |  |
| Fixed effects |  |  |  |  |  |  |  |  |  |  |  |  |  |
| Intercept | 0.021 | 0.001 | **0.001** | 0.020; 0.023 | 0.029 | 0.002 | **0.001** | 0.024; 0.034 |  |  |  |  |  |
| Group status* | -0.003 | 0.001 | **0.001** | -0.004; -0.002 | -0.004 | 0.001 | **0.001** | -0.006; -0.003 |  |  |  |  |  |
| lag1intercept | 0.026 | 0.028 | 0.372 | -0.051; 0.067 | 0.019 | 0.028 | 0.530 | -0.057; 0.057 |  |  |  |  |  |
| Random effects |  |  |  |  |  |  |  |  |  |  |  |  |  |
| Intercept | 2.754^-5^ | 4.864^-6^ | **0.001** | 2.600^-5^; 4.498^-5^ | 2.228^-5^ | 4.857^-6^ | **0.001** | 2.081^-5^; 4.925^-5^ |  |  |  |  |  |
| lag1intercept | 0.008 | 0.006 | 0.456 | 0.009; 0.034 | 0.011 | 0.006 | 0.202 | 0.011; 0.036 |  |  |  |  |  |
| **Acrophase** |  |  |  |  |  |  |  |  |  |  |  |  |  |
| Fixed effects |  |  |  |  |  |  |  |  |  |  |  |  |  |
| Intercept | 14.494 | 0.496 | **0.001** | 13.609; 15.604 | 15.843 | 0.696 | **0.001** | 14.564; 17.230 | 13.804 | 0.488 | **<0.001** | 13.034; 14.940 |  |
| Group status* | 0.256 | 0.096 | **0.004** | 0.072; 0.430 | 0.200 | 0.119 | 0.072 | -0.018; 0.463 | 0.015 | 0.059 | 0.785 | -0.104; 0.128 |  |
| lag1intercept | 0.006 | 0.034 | 0.869 | -0.069; 0.066 | 0.002 | 0.034 | 0.952 | -0.071; 0.061 | 0.013 | 0.035 | 0.755 | -0.067; 0.067 |  |
| Chronotype |  |  |  |  |  |  |  |  | 0.181 | 0.029 | **<0.001** | 0.123; 0.239 |  |
| Random effects |  |  |  |  |  |  |  |  |  |  |  |  |  |
| Intercept | 0.594 | 0.229 | **0.019** | 0.109; 1.036 | 0.594 | 0.245 | **0.022** | 0.047; 1.054 | 0.404 | 0.211 | 0.066 | 0.009; 0.972 |  |
| lag1intercept | 0.001 | 0.001 | 0.430 | 2.572^-5^; 0.005 | 0.001 | 0.001 | **0.412** | 0.001; 0.006 | 0.002 | 0.001 | 0.245 | 0.001; 0.005 |  |

* - depressed group was defined as having a depression diagnosis one month before the assessment

Note. Model 1 is an unadjusted model without covariates. Model 2 adjusted for age, gender, marital status, employment, education, BMI, smoking status, medication use (antidepressants use, benzodiazepines use, antipsychotics use, and lithium use). Model 3 adjusted for chronotype.
